# Case Report: A Novel Homozygous Variant of the *CTSK* Gene in Rare Pycnodysostosis

**DOI:** 10.3390/ijms252313025

**Published:** 2024-12-04

**Authors:** Irina Zhargalovna Zhalsanova, Elizaveta Alekseevna Fonova, Nail Raushanovich Valiakhmetov, Nikita Aleksandrovich Kolesnikov, Sofia Nikolaevna Gosudarkina, Anna Aleksandrovna Agafonova, Ekaterina Georgievna Ravzhaeva, Gulnara Narimanovna Seitova, Vadim Anatolyevich Stepanov, Nikolay Alekseevich Skryabin

**Affiliations:** Research Institute of Medical Genetics, Tomsk National Research Medical Center, Tomsk 634050, Russia; irina.zhalsanova@medgenetics.ru (I.Z.Z.); fonova.elizaveta@medgenetics.ru (E.A.F.); valiakhmetov.nail@medgenetics.ru (N.R.V.); nikita.kolesnikov@medgenetics.ru (N.A.K.); sophia.gosudarkina@medgenetics.ru (S.N.G.); anna.agafonova@medgenetics.ru (A.A.A.); ekaterina.ravzhaeva@medgenetics.ru (E.G.R.); gulnara.seitova@medgenetics.ru (G.N.S.); vadim.stepanov@medgenetics.ru (V.A.S.)

**Keywords:** pycnodysostosis, *CTSK*, bone fragility, next-generation sequencing

## Abstract

Pycnodysostosis (PD) is a rare autosomal recessive skeletal dysplasia from impaired bone resorption due to osteoclastic dysfunction. The features of PD are deformity of the skull, maxilla, and phalanges; osteosclerosis; and bone fragility. We describe the case of a patient with complaints of multiple fractures of the lower extremities in the anamnesis and pain in the lower extremities, cervical spine, and shoulder girdle during physical exertion. Genetic testing revealed a novel homozygous variant c.704T>C (p.Leu235Pro) in the *CTSK* gene. Biallelic pathogenic variants in this gene lead to PD. Thus, the diagnosis in the patient was established by finding a novel likely pathogenic variant in the *CTSK* gene.

## 1. Introduction

Pycnodysostosis (OMIM: 265800) is an ultra-rare osteosclerotic skeletal disorder with an autosomal recessive inheritance type. PD prevalence is between 1 and 1.7 per million population [1].

PD is characterized by facial dysmorphia, osteosclerosis, and bone fragility. Symptoms include short stature, frontal and occipital protrusion, micrognathia, prominent nose, narrow palate, dental anomalies (delayed eruption of primary or permanent teeth, hypodontia, and carious teeth), aplastic clavicle, and skeletal abnormalities such as scoliosis, spondylolysis, spondylolisthesis, brachydactyly, and osteolytic defects of the distal phalanges of the hand. Lifespan, intelligence, and sexual development are not affected. The genetic basis of the disease is due to damage to the *CTSK* gene.

This paper describes a new variant of the *CTSK* gene in a patient with PD.

## 2. Case Presentation

The patient, a 39-year-old woman, presented to the Genetics Clinic with the main complaints of multiple fractures of the lower limbs and pain in the lower limbs, cervical spine, and shoulder girdle during physical activity. Since the age of 12, the patient has been examined by an endocrinologist with a diagnosis of stunted growth due to congenital bone pathology. On the basis of the clinical picture, pycnodysostosis was suspected. Since 8-years old, she had suffered six femoral and tibial fractures, which were treated non-operatively (Table 1).

**Figure 1:**
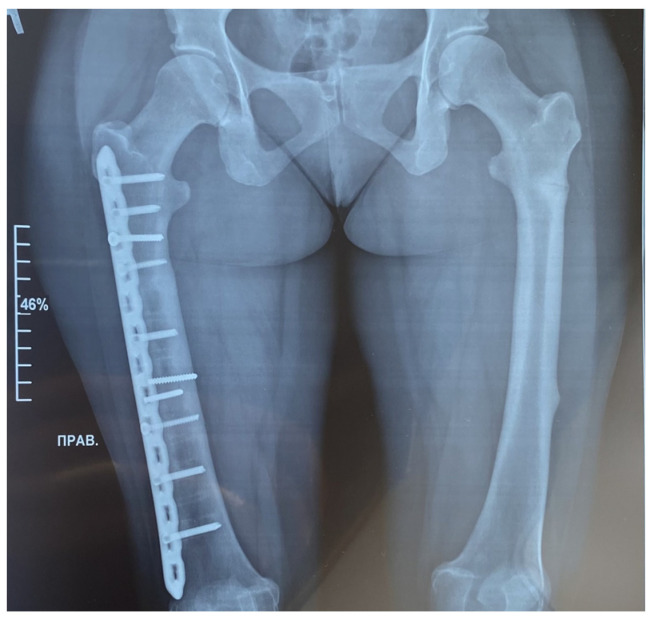
Radiographic examination of the femur bones was conducted using both frontal projections. On the right side, a fracture of the femur was treated with the insertion of a metal osteosynthesis structure (MOS). The position of the metal structure is satisfactory, without displacement. The fracture line is not clearly discernible, yet sufficient consolidation has occurred. Transverse shadows of the previously installed MOS are visible. The axis of the limb remains unchanged. A transverse fracture without displacement is evident on the left side of the diaphysis on the posterior–external surface. The fracture line is clearly visible. Consolidation is poorly expressed. The femur bones show a pronounced thickening of the endosteum, reaching a maximum of 9 mm (bone canal narrowed).

All the fractures were caused by low-energy trauma. At the age of 30, she experienced a closed fracture of the middle third of the left tibia. Surgical treatment was performed: open reposition with correction of the deformity and metal osteosynthesis of the tibia. At the age of 32, she experienced a closed transverse fracture of the middle third of the right femur with displacement of fragments. Surgical treatment was performed using open reposition and osteosynthesis of the femoral fracture with a plate. At the age of 37, she experienced a refracture at the exact same location as the previous one with displacement of fragments. She was treated surgically by open reduction and internal fixation with a ten-hole plate and screws (Figure 1).

**Figure 2:**
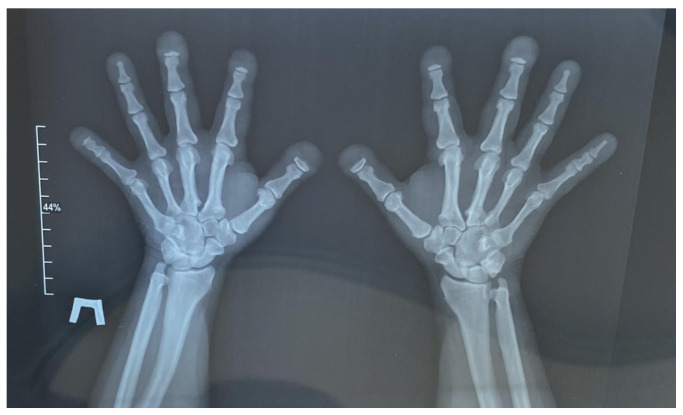
Radiograph of the hands in two projections. Acro-osteolysis with a predominant lesion of the distal phalanges of the 1st, 2nd, 3rd, and 5th digits on the right and the 1st, 2nd, and 3rd digits on the left by the type of nail tubercle resorption is noted. The small tubular bones of the hands are shortened. The interphalangeal and metacarpophalangeal joints are not constricted, and the articular surfaces have clear, even contours. Elongation of the ulnar styloid processes is noted. Para-articular tissues are not altered. There is no evidence of bone destruction.

The patient presented with typical craniofacial dysmorphisms, short stature, and increased bone density. Physical examination affirmed a 150 cm stature, frontal bossing, blue sclera, narrow palate, oligodontia, anomaly of the shape and position of the teeth, four missing lower incisors and one missing lower premolar, and a retrognathic mandible. Hypo-plastic nails and osteolytic defects of the distal phalanges of the hand were also noted. Hand radiographs showed partial dysplasia of the distal phalanx, clinically evident as malformed and dysmorphic fingers (Figure 2).

The standard biochemical parameters were all within normal range, and the alkaline phosphate level was normal. According to densitometry data, bone tissue density was increased.

**Figure 3:**
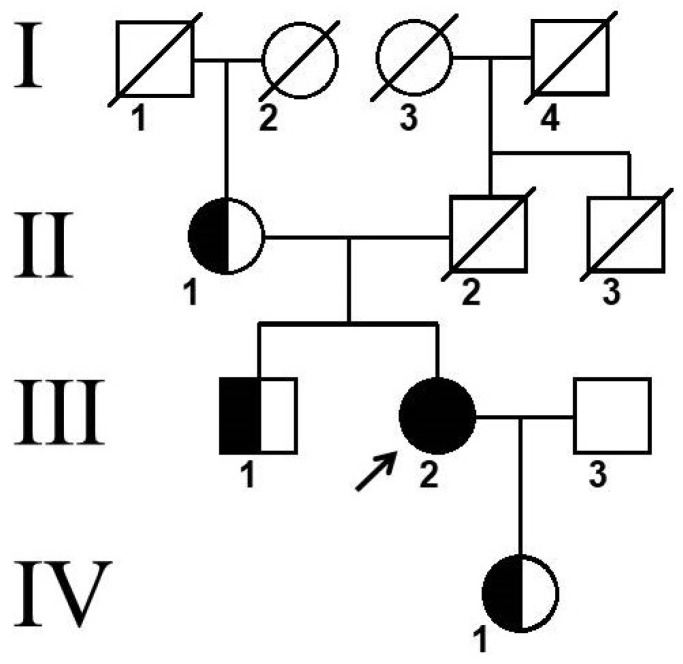
Pedigree of patient. Arrow indicates proband, Roman numerals indicate family generations, and Arabic numerals indicate family members.

The variant c.704T>C (p.Leu235Pro) in the *CTSK* gene was identified in the proband’s mother (II-1), brother (III-1), and daughter (IV-1) in a heterozygous state (Figure 3). Unfortunately, it was not possible to perform genetic analyses in the father’s relatives. According to the family tree, similar symptoms (frontal bossing, short stature) were observed in the father, father’s brother (II-3), and grandfather (I-4).

### 2.1. Massively Parallel Sequencing

**Figure 4:**
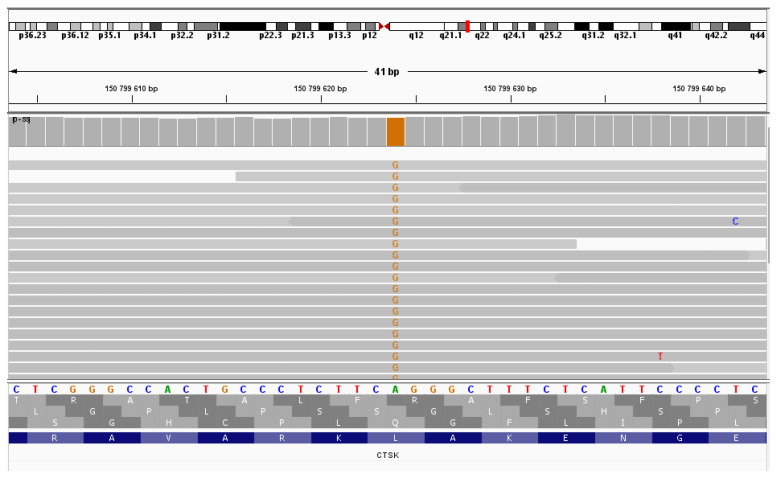
NGS sequencing (IGV browser). Proband sequencing—the variant chr1:150799624A>G in the homozygous state.

Whole exome sequencing of the patient’s DNA revealed the chr1:150799624A>G (NM_000396.4:c.704T>C (p.Leu235Pro)) variant in the *CTSK* gene in the homozygous state (Figure 4). This variant has not been previously described. This nucleotide sequence variant has not been registered in the gnomAD database. The variant is located in a highly conserved position. In silico algorithms predict the pathogenic effect of the variant on the protein. Therefore, according to the ACMG criteria, this variant of the nucleotide sequence variant is regarded as likely pathogenic (PP3, PM2, PP2).

### 2.2. Sanger Sequencing

**Figure 5:**
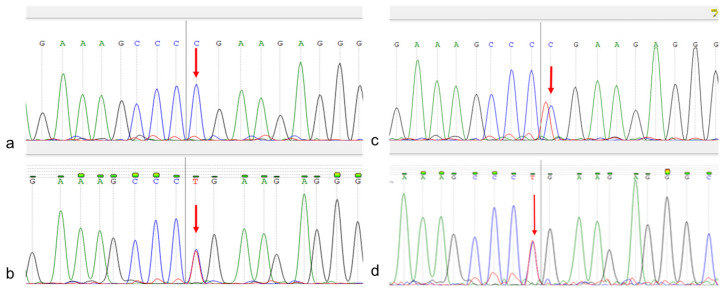
Patient Sanger sequencing. (**a**)—patient; (**b**)—patient’s mother; (**c**)—sibling; (**d**)—patient’s child. The target nucleotide is indicated by an arrow.

Sanger sequencing confirmed the c.704T>C mutation in the patient as homozygous, and that in the patient’s mother, sibling, and child as heterozygous (Figure 5).

### 2.3. Likelihood Ratio (LR) for Relative Pairs Using Autosomal STRs

We checked the frequency of this variant in the population to understand how rare the variant is. A search for carriage of the identified variant was performed in the population whole-genome data of 485 individuals. This variant was not found among the analyzed genomes.

Even though the proband’s parents deny any familial relationship, their carriage of an ultra-rare likely pathogenic variant indicates the possibility of such a relationship. The proband’s father is absent, so segregation analysis is not possible. To test the degree of kinship, we used a system of microsatellite markers. Based on multiplex PCR analysis, data were obtained for 21 autosomal highly polymorphic genomic DNA loci: D3S1358, TH01, D12S391, D5S818, TPOX, D2S441, D7S820, D13S317, FGA, D22S1045, D18S519, D18S519 CSF1PO, D6S1043, vWA, D21S11, SE33, D10S1248, D1S1656, D19S433, and D2S1338. To calculate the degree of relationships, the obtained data were loaded into the Familias program. We compared the performance when considering allele sequences and corresponding putative allele lengths using autosomal STR markers. We evaluated the weight of the evidence for a certain relationship with the likelihood ratio (LR), which involves a comparison of the probabilities of the DNA profiles under two alternative hypotheses (Hp and Hd). LRs < 1 indicate that the genetic evidence suggests that the putative family member is less biologically related.

The paternal genotype was reconstructed for 17 STR loci (due to the presence of a sib); assuming that for 7 loci one allele was guaranteed to be known, the second allele was selected as either similar (homozygote) or alternative, but not from those genotyped in the mother. Such a reconstruction of the paternal genotype can underestimate the LR values, but not overestimate them. The hypothesis was tested that the parents are second cousins compared to random unrelated pairs in the population. A higher LR value between the parents compared to random unrelated pairs from the population may indicate a closer relationship, but is not a guaranteed confirmation of the hypothesis due to unspecified thresholds for its acceptance, a sufficiently limited number of STRs, and the impossibility of accurately reconstructing the paternal genotype. Testing the hypotheses about a cousin and closer relationship between the parents did not show significant differences from random unrelated pairs from the population. The results of the likelihood ratio (LR) and Identical-By-State (IBS, the presence of the same nucleotide sequence) analysis are presented in Table 2.

## 3. Discussion

Pycnodysostosis is a hereditary disease belonging to the group of lysosomal storage diseases, accompanied by compaction of the bone tissue, increased bones fragility, and cranioclavicular dysostosis, and was first described by Maroteaux and Lamy in 1962 [2]. Symptoms of this condition include patients’ short stature, characteristic facial changes (enlarged nose, hypoplasia of the mandible, protruding occipital and frontal tubercles), and frequent pathological fractures.

The genetic basis of the disease is due to damage to the cathepsin K gene (*CTSK*). Cathepsin K is a member of the papain family of cysteine proteinases, expressed exclusively in osteoclasts, which are essential for bone remodeling, repair, and maintenance [3]. This protease plays an important role in osteoclast-induced bone resorption and is responsible for the degradation of type 1 collagen, which makes up 95% of the organic bone matrix. The bones of people with PD are abnormally dense and brittle as a result of this deficient resorption process. [4].

The *CTSK* gene is located on chromosome 1q21, is 14,698 bases in size, and contains eight exons and seven introns. According to ClinVar DB (as of September 2024), there are 113 pathogenic/likely pathogenic variants in the *CTSK* gene, including 42 frameshift, 26 missense, 26 nonsense, and 19 splice-site variants [5].

Pycnodysostosis is classified in the same group as osteopetrosis in the international classification of hereditary skeletal diseases [6]. Osteopetrosis is also characterized by generalized osteosclerosis. Due to the similarity in clinical manifestations, an early differential diagnosis is crucial, as the approaches to treatment utilizing molecular genetic techniques are different.

The predisposition to tubular bone fractures in older age constitutes a key specific feature, which is of importance for its diagnosis. Previous research has demonstrated that the first bone fractures in PD patients most commonly occur between the ages of 7 and 10 years and are characterized by slow healing [7].

This study describes clinical presentation of a patient with multiple fractures (the first fractures were recorded at the age of 8 years) of the lower extremities, short stature, osteolytic defects of the distal phalanges of the upper and lower extremities, prominent frontal and occipital protuberances, hypoplasia of the mandible, narrow hard palate with a cleft, and oligodontia. The endocrinologist made a preliminary diagnosis of PD and the patient was sent for genetic analysis to search for a genetic variant.

The necessity for prompt diagnosis of pycnodysostosis is predominantly contingent upon the distinctive characteristics of the therapeutic intervention. Consequently, patients diagnosed with pycnodysostosis are explicitly excluded from bisphosphonate therapy, which is a mandatory requirement for patients with osteopetrosis.

Whole exome sequencing of the patient’s DNA revealed a homozygous c.704T>C (p.Leu235Pro) variant in the *CTSK* gene. Sanger sequencing confirmed the homozygous variant in the patient and the heterozygous variant in the patient’s mother, sibling, and child.

This variant has not been previously described. In the literature, 70 percent of mutations were shown to be found in the mature domain, similar to the identified variant [8]. In addition, the vast majority of pathogenic and likely pathogenic variants in the gene are missense mutations [5]. A previously described pathogenic variant, c.746T>A (p.Ile249Asn), in the homozygous state and associated with pycnodysostosis is located close to identified variant. Extensive modeling of the protein sequence and biophysical properties (such as structural, functional and spatial information, amino acid conservation, physicochemical variability, residue mobility, and thermodynamic stability) performed in Invitae indicate that this missense variant is expected to disrupt the function of the CTSK protein [9]. Another previously described pathogenic variant, c.926T>C (p.Leu309Pro), which also involves a substitution of leucine for proline, was observed in patients with pycnodysostosis. The authors describe that the variant is located in a highly conserved region and is likely to cause disease [10]. The variant we identified is also located in a conservative region. We performed multiple sequence alignment in different species: *Danio rerio*, *Mus musculus*, *Gallus gallus*, *Pan troglodytes*, and *Homo sapiens*. The results confirmed the conservatism of the region, which indicates the pathogenicity of the variant (Table 3).

According to the literature data and ACMG criteria, this variant of the nucleotide sequence variant is regarded as likely pathogenic.

It is known that 20% of children are born in consanguineous marriages [11]. The proband’s mother states that there is no consanguineous marriage. In our case, however, it is impossible to conduct segregation analysis due to the absence of the proband’s father. Also, according to the patient, her father’s relatives had symptoms of a possible disease. In this regard, we decided to test the carriage of this variant in our sample of whole-genome data from 485 people. Analysis of the degree of kinship between the proband’s parents showed that LR was higher than in other random unrelated pairs in the population (Table 1), which may indicate a closer relationship between the proband’s parents.

## 4. Materials and Methods

### 4.1. Massively Parallel Sequencing and Sanger Sequencing

DNA was isolated from whole blood using phenol–chloroform extraction. The patient’s DNA was analyzed using a next-generation sequencer, GeneMind GenoLab (GeneMind, Shenzhen, Guangdong, China). Targeted enrichment using an Agilent Sure Select All Exon v7 kit (Agilent, Santa Clara CA, USA) was used for sample preparation. Sequencing data were analyzed using the GATK Best Practices Germline short variant discovery pipeline. Reads were aligned to the reference human genome sequence (hg38), including alignment post-processing, variant calling, quality filtering, and annotation of the identified variants by the canonical transcript of each gene. Variants that did not meet the quality criteria were excluded from further analysis. The pathogenicity of the variants was determined considering the ACMG recommendations. Reads had a length of 2 × 101 bp. The median reliability of nucleotide determination was higher than Q30. The median coverage was 59×, and the mean coverage was 69.3×, Q30—83.3%, Q10—94.2%.

Sanger sequencing around the identified variant was performed in both directions in the patient and her relatives (establishment of a mutation carrier). The following primers for the PCR were selected: Forward: 5′-CTGACTCATTGCCTATTGCT-3′, Reverse: 5′-ACTGAAAATACCCAAGGTCC-3′. The size of the amplified segment for Sanger sequencing is 305 base pairs.

### 4.2. Multiple Sequence Alignment

Clustal Omega is a widely used package for carrying out multiple sequence alignment, and the accuracy of protein alignments is high when compared to alternative packages [12]. Multiple sequence alignment is generally the alignment of three or more biological sequences (protein or nucleic acid) of similar length. From the output, homology can be inferred and the evolutionary relationships between the sequences studied.

### 4.3. Calculating the Degree of Relationships of the Proband’s Parents

The microsatellite marker system of the COrDIS “EXPERT 26” kit (Gordiz, Moscow, Russia) was used to assess the degree of relationship of the studied DNA samples between the proband’s relatives. The PCR products of individual loci were separated on a NANOFOR-05 genetic analyzer (Synthol, Moscow, Russia) in the presence of DNA length standards S550 (COrDIS) under the conditions recommended by the manufacturer. Fragment size analysis was performed using GeneMarker Software (V.3.0.1) (State College, PA, USA). We used the Familias program to calculate the probability of a relationship and identification based on the DNA data. The Familias program may be used to compute probabilities and likelihoods in cases where the DNA profiles of some people are known, but their family relationship is in doubt. A database of allele frequencies of 20 autosomal STRs generated from a population sample of reference DNA was used.

A sample of 485 individuals from four regions, including indigenous populations of Siberia (74), Eastern Europe (160), the Caucasus (211), and Western Asia (30), was used to search for the identified variant in the genome-wide data. Bcftools v.1.18 was used for data extraction. Bcftools—utilities for variant calling and manipulation of VCFs and BCFs.

## 5. Conclusions

In conclusion, this report expands the list of likely pathogenic variants in the *CTSK* gene that cause pycnodysostosis. Our study demonstrates the utility of whole exome sequencing in the diagnosis of diseases with similar clinical features. In the future, disease-specific treatments to prevent complications and improve patients’ quality of life may be identified through similar studies.

## Figures and Tables

**Table 1:** A list of the patient’s bone fractures.

№	Patient’s Age	Type of Fracture	Bones
1	8 years old	Closed fracture	the left fibula
2	9 years old	Closed fracture	the right fibula
3	10 years old	Closed fracture	the tarsal bones of the right foot
4	12 years old	Repeated closed fracture	the right tarsal bones
5	16 years old	Repeated closed fracture	the left fibula
6	20 years old	Closed fracture	the right tibia
7	30 years old	Closed fracture	the middle third of the left tibia
8	32 years old	Closed transverse fracture with displacement of fragment	the middle third of the right femur
9	37 years old	Closed peri-implant fracture with displacement of fragment	the right femur

**Table 2:** Likelihood ratio and Identical-By-State analysis for relative pairs.

Person 1	Person 2	Relationship	LR	IBS = 2	IBS = 1	IBS = 0
D9119	D9118_dg3	2nd Cousins	8.44854	6%	41%	53%
D9119	D9118_dg1	2nd Cousins	8.19546	6%	41%	53%
D9119	D9118_dg4	2nd Cousins	6.84144	6%	41%	53%
D9119	D9118_d3	2nd Cousins	6.81609	0%	47%	53%
D9119	D9118_dg2	2nd Cousins	6.6365	6%	41%	53%
D9119	D9118_d1	2nd Cousins	6.61191	0%	47%	53%
D9119	D9118_d4	2nd Cousins	5.51952	0%	47%	53%
D9119	D9118_d2	2nd Cousins	5.35418	0%	47%	53%
RUS42	RUSk77	2nd Cousins	4.78296	6%	65%	29%
RUSk26	RUSk66	2nd Cousins	4.49137	13%	56%	31%
RUSk4	RUSk7	2nd Cousins	4.43016	12%	65%	24%
RUS57	RUSk64	2nd Cousins	4.02469	6%	47%	47%
RUS15	RUSk46	2nd Cousins	3.70891	18%	59%	24%
RUS63	RUSk53	2nd Cousins	3.54992	6%	81%	13%
RUSk2	RUSk7	2nd Cousins	3.50763	12%	53%	35%
RUS9	RUSk76	2nd Cousins	3.4613	18%	47%	35%
RUS32	RUSk40	2nd Cousins	3.37673	18%	59%	24%
RUS39	RUSk52	2nd Cousins	3.31093	18%	65%	18%
RUSk45	RUSk63	2nd Cousins	3.24991	13%	73%	13%
RUS16	RUSk38	2nd Cousins	3.14799	12%	47%	41%
RUS8	RUS16	2nd Cousins	3.10237	12%	59%	29%
RUS59	RUSk36	2nd Cousins	3.05579	12%	59%	29%
RUSk46	RUSk47	2nd Cousins	3.00883	18%	47%	35%

Note: LR—likelihood ratio. IBS—Identical By State (total number possible shared for the overlapping markers): IBS = 0 (no overlapping markers); IBS = 1 (overlapping markers with 1 allele); IBS = 2 (overlapping markers with 2 alleles).

**Table 3:** Multiple sequence alignment.

Organism	NCBI Gene ID	Sequence Around the Variant	Sequence Coordinates
*Danio rerio*	NC_007127.7: 29492994–29502549 ctsk	––––G**C**AGAGAA	NC_007127.7: 29497635–29497647
*Mus musculus*	NC_000069.7: 95406521–95416698 Ctsk	ATCAG**G**AAAGTA	NC_000069.7: 95409315–95409327
*Gallus gallus*	NC_052556.1: c2553581–2550760 CTSK	–––––––––––––––––	0
*Pan troglodytes*	NC_072398.2: 98404833–98418903 CTSK	ATCAG**C**AAGATA	NC_072398.2: 98410255–98410267
*Homo sapiens*	NC_000001.11: c150808260–150796208 CTSK	ATCAG**C**AAGATA	NC_000001.11: 150799619–150799631

## Data Availability

Due to privacy policies, data are available on request after the approval of the patients.
